# Study of the frequency of *Clostridium difficile tcdA, tcdB, cdtA* and *cdtB* genes in feces of Calves in south west of Iran

**DOI:** 10.1186/1476-0711-13-21

**Published:** 2014-06-05

**Authors:** Abbas Doosti, Abbas Mokhtari-Farsani

**Affiliations:** 1 Biotechnology Research Center, Islamic Azad University, Shahrekord Branch, Postal Box: 166, Shahrekord, Iran

**Keywords:** *Clostridium difficile*, Calves, Toxin A, Toxin B, Binary toxin

## Abstract

**Background:**

*Clostridium difficile* (*C. difficile*) is a gram-positive, toxin-producing bacillus which is an intestinal pathogen in both humans and animals and causes a range of digestive disorders including inflammation of the bowel, abdominal pain, fever and diarrhea. *C. difficile* toxins include enterotoxin (Toxin A), cytotoxin (Toxin B) and a binary toxin. Two large protein toxins A and B are encoded by separate genes, *tcdA* and *tcdB. Clostridium difficile* infection (CDI) mainly caused by the activity of the genes *tcdA* and *tcdB*. The binary toxin is encoded by the genes *cdtA* and *cdtB.* The binary toxin caused increased adherence of bacteria to intestinal epithelium. The aim of the present study was isolation of *C. difficile* from feces of calves, and study of the frequency of *C. difficile* virulence genes.

**Methods:**

150 samples of fresh feces from calves were collected and *C. difficile* was isolated from feces of calves using bacterial culture methods. DNA was extracted by a genomic DNA purification kit. Then PCR method was used for definitive diagnosis of *C. difficile*. Multiplex PCR method performed for identification of *tcdA*, *tcdB*, *cdtA* and *cdtB* genes. In the final stage antimicrobial resistance determining was carried out by standard Bauer-Kirby disk diffusion method.

**Results:**

*C. difficile* was isolated from 90 samples (60%). The *tcdA* was observed in 8 isolates (8.8%), *tcdB* in 16 isolates (17.7%), *cdtA* in 8 isolates (8.8%) and *cdtB* in 14 isolates (15.5%). Only 1 isolated (1.1%) was containing all four genes *tcdA*, *tcdB*, *cdtA* and *cdtB*, 2 isolates (2.2%) only had both *tcdA* and *tcdB* genes, and there was no sample positive only for both *cdtA* and *cdtB*. The highest rate of drug resistance was against clindamycin (100%) and the highest rate of drug sensitivity was against ciprofloxacin (50%).

**Conclusion:**

The results showed high incidence of *C. difficile* and also high antibiotic resistance of this bacterium, but frequency of strains containing virulence genes (*tcdA*, *tcdB*, *cdtA* and *cdtB*) was low.

## Background

*Clostridium difficile (C. difficile)* is a gram-positive, spore-forming, anaerobic, toxin-producing bacillus, catalase-negative bacterium and is part of the normal gut flora in less than 5% of humans that can infect both humans and animals [1]. *C. difficile* there at everywhere in the environment and as well as exist as free-living bacteria that it has been found in farm animals, pets, on various surfaces in hospitals and in foods such as vegetables, meats, water and soil [2]. It is transmitted via the fecal–oral route among humans and also is transmitted to humans from animals and animal products. *Clostridium difficile* infection (CDI) is a global problem that is caused by the ingestion of vegetative organisms and spores, most likely the latter which survive exposure to gastric acidity and germinate in the colon. *C. difficile* recognized as a major nosocomial pathogen responsible for 15-20% of antibiotic-related diarrhea and is the etiologic agent of perforation of the colon, pseudomembranous colitis or toxic megacolon and even death in humans [3,4]. *C. difficile* also is an important factor for of enteric disease in other species, including calves (10.2%), cows, ostriches, horses, pigs, rabbits, cats and dogs [5,6]. The symptoms of CDI in both humans and animal include inflammation of the bowel, abdominal pain, fever and diarrhea [7]. Recently, research has suggested that the frequency, relapse, and severity of *Clostridium difficile* associated diseases (CDAD) are increasing in North America and Europe and also per year 2012, United States health care expenses associated with treating CDAD are estimated about $3.2 billion [8]. *C. difficile* toxins include toxin A, toxin B and a binary toxin. Toxins A (enterotoxin) and B (cytotoxin) (308 kDa and 270 kDa, respectively), are encoded by two separate genes, *tcdA* and *tcdB* which are located in a 19.6-kb pathogenicity locus (PaLoc) and are responsible for the symptoms of CDAD and also includes the positive and negative regulators *tcdR* and *tcdC*[9]. They are structurally similar to each other, but toxin B is generally more robust (~1000 fold) than toxin A [10]. The binary toxin is encoded by the genes *cdtA* and *cdtB* located outside PaLoc, which these two genes form another operon with *cdtR* (*cdtR* is a positive regulator) [9]. *C. difficile* toxins directly affect the colon epithelial cells, and immune cells are forced to produce chemokines and cytokines [10]. Standard treatment for CDI in both humans and animals is use of the vancomycin or metronidazole which neither of these antibiotics is not fully effective, but new therapeutic options include the use of new antibiotics, probiotics, toxin-absorbing polymer, monoclonal antibodies, fecal treansplant, toxoid vaccines and Intravenous immunoglobulin (IVIG) [10]. To our knowledge, there are no many studies on *C. difficile* in feces calves in around the world and also there are no study on *C. difficile* in feces calves in Iran; thus, the purpose of present study was isolation of *C. difficile* from feces of calves from Chaharmahal va Bakhtiari province in south west of Iran using bacterial culture methods, and study of the frequency of *C. difficile tcdA, tcdB, cdtA* and *cdtB* genes using Multiplex PCR method, and also study of antibiotic resistance in this bacterium.

## Methods

### Samples collection

In this cross-sectional descriptive study, a total one hundred fifty (150) samples of fresh feces from calves with age 3 to 25 days in autumn of 2013 were collected from industrial and indigenous calves farms in Chaharmahal va Bakhtiari province (southwest of Iran). Only one stool sample was collected from each calf. Feces were collected from animals rectum using the new clean gloves and about 3 g of the sample was transferred to a new sterile Tube and were transported to the laboratory immediately in an insulating foam box with ice.

### *C. difficile* Culture

**Figure 1:**
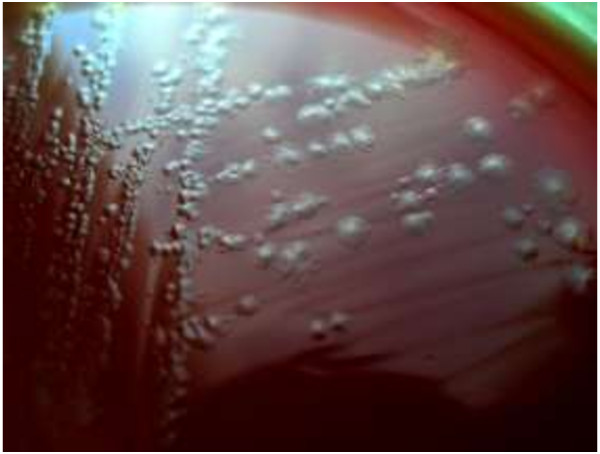
***C. difficile *** **colony morphology.**

Outset was placed tubes containing 10 ml cooked meat broth medium (Merckoplate, Germany) at 80°C in a water bath for 10 min until the medium be anaerobic. Then it was placed at room temperature until be cooled and about 2 g of feces were inoculated into it and was placed at 65°C in a water bath for 10 min in order to select bacterial spores and was incubated in an anaerobic incubator (Finetech, Germany) at 37°C for 7 days. Thereafter, removed from the suspension by a sterile loop and was cultured onto blood agar supplemented with 5% sheep blood (Merckoplate, Germany) to linear method and was incubated in an anaerobic incubator at 37°C for 48 h. Identification and isolation of *C. difficile* was based on the characteristic morphology, gram stain and the smell of the colonies. The colonies circular, tarnishes, slightly embossed, white or gray was indicative the presence of *C. difficile* (Figure 1). In order to keep the bacteria for late experiments, the plates were wrapped in parafilm and were kept at 4°C.

### DNA extraction and PCR

*C. difficile* was grown on blood agar for 48 h and approximately 3 colonies were suspended in 100 μL of sterile ultrapure deionized water and DNA was extracted by a genomic DNA purification kit (CinnaGen Co, Iran) according to the manufacturer’s instructions. The total DNA was measured at 260 nm optical density according to the method described by Sambrook and Russell (2001). DNA samples were stored at −20°C until they were used. Polymerase chain reaction (PCR) method was used for definitive diagnosis of *C.difficile*. Detection of *C. difficile* was performed by amplification with the following primers: C.D-F: 5’-CCTCCTCAAGTACCGTCATTATC-3’ and C.D-R: 5’-CAAAGGTGAGCCAGTACAG GA-3’ [Gene Bank: NC_009089 ]. The primers were design from 16S ribosomal RNA (16S rRNA) gene of *C. difficile* using Gene Runner software (Version 3.01) and at the NCBI using the experimental GENINFO BLAST Network Service to assess degree of homology between these primers and other reported sequences and at the end were obtained from CinnaGen Co, Iran. PCR reactions were performed in a total volume of 25 μL in 0.2 ml tubes containing 2 μL of DNA sample, 1 μM of each primers, 2 mM MgCl2 , 5 μL of 10X PCR buffer AMS, 200 μM dNTPs and 1 unit of Taq DNA polymerase (CinnaGen Co, Iran). The PCR assay was performed at 95°C for 5 min and then for 32 cycles of 94°C for 1 min, 61°C for 40 sec, 72°C for 40 sec, and a final extension at 72°C for 5 min, with a final hold at 10°C in a thermal cycler (Mastercycler gradient, Eppendrof, Germany).

### Detection of PCR products

The PCR-amplified products (C.D 16S rRNA, 264 bp) were detected in 1.5% ethidium bromide (EtBr)-stained agarose gel electrophoresis. Constant voltage of 84 V for 25 min was used for products separation. The DNA molecular weight marker (100 bp, Fermentas, Germany) was used as a size marker. The gel viewed on UV transilluminator and photographed were obtained in UVI doc gel documentation systems (UK).

### Multiplex PCR for identification of *tcdA*, *tcdB*, *cdtA* and *cdtB* genes

A 4-plex PCR was performed for identification of *tcdA*, *tcdB*, *cdtA* and *cdtB* genes. All four primers pairs for multiplex PCR were designed and obtained by using the method listed before (Table 1). The multiplex PCR was run in final reaction volumes of 50 μL containing the following reagents: 2 μL of genomic DNA, 200 μM each of dATP, dCTP, dGTP and dTTP, 50 pmol of each primer (primers details are presented in Table 1), 6 mM MgCl2, 50 mM KCl, 10 mM Tris–HCl (pH 8.5) and 5 unit of Taq DNA polymerase (CinnaGen Co., Iran). Reactions were initiated at 95°C for 5 min, followed by 40 cycles of 94°C for 1 min, 59°C for 1 min, 72°C for 1 min and a final extension step at 72°C for 7 min, with a final hold at 10°C in a thermal cycler (Mastercycler gradient, Eppendrof, Germany). Detection of PCR products was performed by using the method listed before.

**Table 1:** **Primers for identification of C. *difficile tcdA, tcdB, cdtA *and *cdtB *genes**

**Gene target**	**Primer name**	**Sequence (5'–3')**	**Accession**	**Amplicon size (bp)**
***tcdA***	tcdA-F tcdA-R	AATGATGTTACCTAATGCTCCTTC	KC292125	311 bp
		AGTAAGTTCCTCCTGCTCCATC		
***tcdB***	tcdB-F tcdB-R	CCAGCTAATACACTTGATGAAAACC	KC292190	432 bp
		TTTCTTCACCTTCTTCATTTCCT		
***cdtA***	cdtA-F cdtA-R	GGAAGCACTATATTAAAGCAGAAGC	HQ639678	219 bp
		TCTGGGTTAGGATTATTTACTGGAC		
***cdtB***	cdtB-F cdtB-R	AAAGTTGATGTCTGATTGGGAAG	HQ639678	608 bp
		TTTGTTGTTGGTGTCACTTTGTA		

### Antimicrobial drug susceptibility tests

Drug resistance testing using standard Bauer-Kirby disk diffusion method for all samples (positive culture) was performed. A total of 5 antibiotic discs (Padtanteb, Iran) with tetracycline, ciprofloxacin, clindamycin, erythromycin and vancomycin were used. These antimicrobial agents were chosen on the basis of their importance in treating humans or animals CDI. Diameters of the inhibition zones were interpreted based on the NCCLS subcommittee’s recommendations [11].

### Statistical analysis

All data were analyzed by using MS Excel 2007 and SPSS software (Version 17.SPSS Inc, USA) and p value was calculated using Chi-square and Fisher’s exact tests to find any significant relationship. P value less than 0.05 was considered statistically significant.

## Results

### The frequency of *C. difficile* and virulence genes

**Figure 2:**
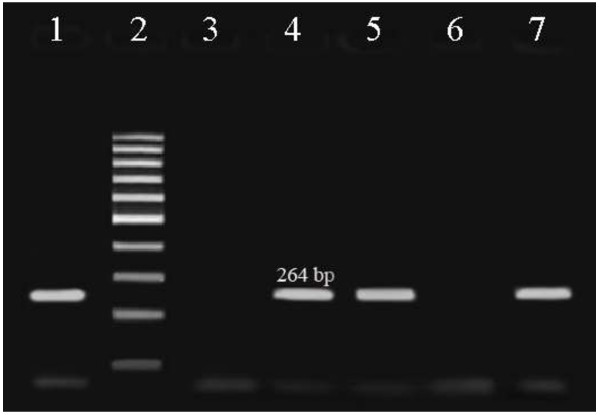
**Gel electrophoresis for detection of *C. difficile *** **(Lane 2 shows fermentas 100 bp DNA molecular marker, Lanes 1, 4, 5 and 7 are positive samples for *C. difficile*, and Lanes 3 and 6 are negative for *C. difficile*).**

**Figure 3:**
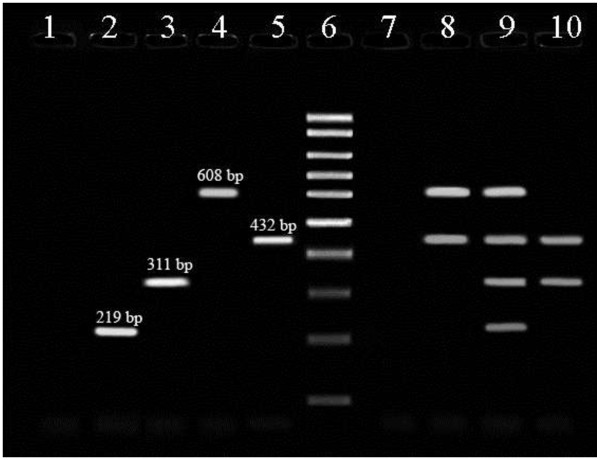
**Multiplex-PCR for detection of virulence genes (Lanes 1 and 7 are negative for virulence genes, Lanes 2, 3, 4 and 5 are positive only for one of virulence genes, Lane 6 shows fermentas 100 bp DNA molecular marker, Lane 8 is positive for *tcdB *** **and *cdtB *** **genes, Lane 9 is positive for all four genes *tcdA*, *tcdB*, *cdtA *** **and *cdtB*, and Lane 10 is positive for *tcdA *** **and *tcdB *** **genes).**

In the present study, a total of 150 samples of feces of calves were tested for *C. difficile*. In order to diagnosis of *C. difficile*, PCR test was carried out. PCR products on 1.5% agarose gel showed 264 bp fragment for *C. difficile* (Figure 2). The results obtained revealed that there are 90 samples (60.0%) containing *C. difficile*, and also it was found that the peak incidence of *C. difficile* in calves is in age 16 days (21.3%). A 4-plex PCR was developed for study of the frequency of virulence genes (*tcdA*, *tcdB*, *cdtA* and *cdtB*) (Figure 3). Eight isolates (8.8%) possessed the *tcdA* gene, sixteen isolates (17.7%) possessed the *tcdB* gene, eight isolates (8.8%) possessed the *cdtA* gene and fourteen isolates (15.5%) possessed the *cdtB* gene. Two isolates (2.2%) were positive only for both *tcdA* and *tcdB* genes, three isolates (3.3%) were positive only for both *tcdB* and *cdtB* genes and only one isolated (1.1%) was containing all four genes *tcdA*, *tcdB*, *cdtA* and *cdtB*, in addition, there was no sample positive only for both *cdtA* and *cdtB*.

### Antibiogram profile

The resistance pattern of *C. difficile* isolates to 5 antimicrobial agents tested in this study is shown in Table 2. All isolates (100%; n = 90) were resistant to one or more antimicrobial agent. The highest rate of drug resistance was against clindamycin (100%) and erythromycin (90.0%), in addition, the highest rate of drug sensitivity was against ciprofloxacin (50.0%) and vancomycin (20.0%). Three isolates (3.3%) were only resistant to single antibiotic, fifteen isolates (16.6%) were resistant to 2 antibiotics, forty five isolates (50.0%) were resistant to 3 antibiotics and twenty seven isolates (30.0%) showed resistance to 4 or more than 4 antimicrobial agents.

**Table 2:** **Antibiogram profile of the 90 *C. difficile *isolates from feces of calves**

**Antibiotic discs**	**Sensitivity**		**Intermediate**		**Resistance**	
	**No. of samples positive**	**% of samples positive**	**No. of samples positive**	**% of samples positive**	**No. of samples positive**	**% of samples positive**
Ciprofloxacin (5 μg)	45	50	15	16.6	30	33.3
Erythromycin (15 μg)	0	0	9	10	81	90
Clindamycin (2 μg)	0	0	0	0	90	100
Tetracycline (30 μg)	15	16.6	69	76.6	6	6.6
Vancomycin (2 μg)	18	20	0	0	72	80

## Discussion

*C. difficile* was recognized as an intestinal pathogen in the late 70th of twentieth century [12]. *C. difficile* is agent for more than 95% of cases of pseudomembranous colitis and 15–25% of cases of antibiotic-associated diarrhea [13]. CDAD in humans may have an animal origin, for example, *C. difficile* is found in market meat, and studies have shown that Pathogenic strains in humans and animals especially calves, are very similar [6]. In recent years, has emerged changes in the epidemiology of CDAD in humans and also CDI is increasing in prevalence, severity and mortality. Development of antibiotic resistance in *C. difficile* serotypes has been considered as a public health problem throughout the world. It seems that in order to determine the epidemiology of *C. difficile*- associated disease should studies and more research be done around the world. In present study was observed a relatively high prevalence of *C. difficile* and virulence genes, and also isolates showed high percentage of resistance against antibiotics. The study of Arroyo et al. showed that *C. difficile* isolated from calves are carrier the genes encoding toxins A and B [14]. In Rodriguez-Palacio et al. investigation in Canada, contamination rate with *C. difficile* in calves with a mean age of 14.2 days, 11.2% was reported, also examined *C. difficile* ribotypes, which 7 of 8 ribotype were common between humans and calves, furthermore, in antibiotic susceptibility testing, all isolates were sensitive to vancomycin, which is somewhat similar to the present study results [5]. In Pirs et al. investigation, *C. difficile* was isolated from 1/56 calf samples (1.8%) [6]. The study of Janvilisri et al. showed that only 9 (52.9%) of the 17 calves were positive to *C. difficile* and every 9 positive samples were possessed the binary toxin, which is somewhat similar to the present study results [15]. Hoffer et al. in Switzerland, announced Low occurrence of *C. difficile* in stool of calves, that *C. difficile* was isolated from only one fecal sample of a calf, furthermore, was determined that sample isolated is containing toxin A, toxin B and binary toxin [16]. In Rodriguez et al. investigation in Belgium, prevalence of *C. difficile* was reported in calves on the farms 22.2% [17]. Zidaric et al. found that the peak incidence of *C. difficile* in calves is in age 18 days (16%), also was found that the isolates were resistant to erythromycin, which is similar to the present study results [18].

The above data indicate that in around the world there are different rate of frequency of *C. difficile* and virulence genes of this bacterium. Furthermore, with review previous studies will be determined that there are different patterns of antimicrobial resistance of *C. difficile* isolated of different parts of the world. Our study and other studies throughout the world show that in recent years prevalence of pathogenic strains and resistant to antimicrobial drugs with speed is on the rise. Considering that, there aren’t large studies on virulence genes of *C. difficile* isolated from calves and domestic animals, the prevalence of these genes cannot be examined, but considering that amount of pathogenicity of *C. difficile* in worldwide is rising can be said that the prevalence of pathogenic strains that are containing these genes is on the rise. In the present study it was determined that ciprofloxacin is the best antibiotic for infections treatment caused by *C. difficile* and although there was a high rate of sensitivity to vancomycin, but resistance rate to this antibiotic was 80%. Nonetheless, examining antibiotic resistance patterns in other studies indicate that vancomycin is currently the best antibiotic for infections treatment caused by *C. difficile* in calves and other domestic animals, that is inconsistent with the results of our study.

In conclusion, our data show high prevalence of *C. difficile* in feces of calves in south west of Iran, that fortunately frequency of pathogenic strains (containing virulence genes) was low. On the other hand, our studies showed a high percentage of antibiotic resistance in the *C. difficile* which is a result of the indiscriminate use of antibiotics. Thus, given that many of *C. difficile* strains are common between humans and domestic animals and given the increasing importance of CDI in public health, we suggest further studies for recognition the epidemiology and detect any changes in resistance pattern, new therapies, restricting use of antimicrobial drugs in human and animals, performing antimicrobial susceptibility tests to select suitable antimicrobial agent, application of recommended dosage of antibiotic and following duration of therapy can help to decrease developing of resistant strains and pathogenic.

## Competing interests

The authors declare that they have no competing interests.

## Authors’ contributions

AD: Study supervision, study concept and design, critical revision of the manuscript for important intellectual content. AM-F: Drafting of the manuscript, critical revision of the manuscript for important intellectual content, carried out the molecular genetic studies, analyzed the data. All authors read and approved the final manuscript.

## References

[B1] Hammond EN Donkor ES Antibacterial effect of Manuka honey on *Clostridium difficile* BMC Res Notes 2013 6 1 5 10.1186/1756-0500-6-1 23651562 PMC3669629

[B2] Rogers MAM Greene MT Saint S Chenoweth CE Malani PN Trivedi I Aronoff DM Higher Rates of *Clostridium difficile* Infection among Smokers Plos one 2012 7 e42091 10.1371/journal.pone.0042091 22848714 PMC3407081

[B3] Janvilisri T Scaria J Chang YF Transcriptional profiling of *Clostridium difficile* and Caco-2 cells during infection J Infect Dis 2010 202 282 290 10.1086/653484 20521945 PMC2891111

[B4] Hung YP Tsai PJ Hung KH Liu HC Lee CI Lin HJ Wu YH Wu JJ Ko WC Impact of Toxigenic *Clostridium difficile* Colonization and Infection among Hospitalized Adults at a District Hospital in Southern Taiwan Plos one 2012 7 e42415 10.1371/journal.pone.0042415 22876321 PMC3411658

[B5] Rodriguez-Palacios A Stämpfli HR Duffield T Peregrine AS Trotz-Williams LA Arroyo LG Brazier JS Weese JS *Clostridium difficile* PCR Ribotypes in Calves, Canada Emerging Infect Dis 2006 12 1730 1736 10.3201/eid1211.051581 17283624 PMC3372327

[B6] Pirs T Ocepek M Rupnik M Isolation of *Clostridium difficile* from food animals in Slovenia J Med Microbiol 2008 57 790 792 10.1099/jmm.0.47669-0 18480339

[B7] Davies NL Compson JE MacKenzie B O’Dowd VL Oxbrow AKF Heads JT Turner A Sarkar K Dugdale SL Jairaj M Christodoulou L Knight DE Cross AS Hervé KJ Tyson KL Hailu H Doyle CB Ellis M Kriek M Cox M Page MJ Moore AR Lightwood DJ Humphreys DP A Mixture of Functionally Oligoclonal Humanized Monoclonal Antibodies That Neutralize *Clostridium difficile TcdA* and *TcdB* with High Levels of In Vitro Potency Shows In Vivo Protection in a Hamster Infection Model Clin Vaccine Immunol 2013 20 377 390 10.1128/CVI.00625-12 23324518 PMC3592348

[B8] Swett R Cisneros GA Feig AL Conformational Analysis of *Clostridium difficile* Toxin B and Its Implications for Substrate Recognition Plos one 2012 7 e41518 10.1371/journal.pone.0041518 22844485 PMC3402401

[B9] Persson S Jensen JN Olsen KEP Multiplex PCR Method for Detection of *Clostridium difficile tcdA*, *tcdB*, *cdtA*, and *cdtB* and Internal In-Frame Deletion of tcdC J Clin Microbiol 2011 49 4299 4300 10.1128/JCM.05161-11 21976756 PMC3232998

[B10] Sun X Savidge T Feng H The Enterotoxicity of *Clostridium difficile* Toxins Toxins 2010 2 1848 1880 10.3390/toxins2071848 22069662 PMC3153265

[B11] National Committee for Clinical Laboratory Standards Performance standards for antimicrobial susceptibility testing Twelfth Informational Supplement 2002 Wayne: NCCLS document M100-S12 22, NCCLS 42

[B12] Lemee L Dhalluin A Testelin S Mattrat MA Maillard K Lemeland JF Pons JL Multiplex PCR Targeting tpi (Triose Phosphate Isomerase), *tcdA* (Toxin A), and *tcdB* (Toxin B) Genes for Toxigenic Culture of *Clostridium difficile* J Clin Microbiol 2004 42 5710 5714 10.1128/JCM.42.12.5710-5714.2004 15583303 PMC535266

[B13] Sadeghifard N Salari MH Ranjbar R Ghafouryan S Raftari M Abdulamir AS Fatimah AB Kazemi B The clinical and environmental spread and diversity of toxigenic *Clostridium difficile* diarrhea in the region of the Middle East Reviews in Infect 2010 1 180 187

[B14] Arroyo LG Kruth SA Willey BM Staempfli HR Low DE Weese JS PCR ribotyping of *Clostridium difficile* isolates originating from human and animal sources J Med Microbiol 2005 54 163 166 10.1099/jmm.0.45805-0 15673511

[B15] Janvilisri T Scaria J Thompson AD Nicholson A Limbago BM Arroyo LG Songer JG Grohn YT Chang YF Microarray Identification of *Clostridium difficile* Core Components and Divergent Regions Associated with Host Origin J Bacteriol 2009 191 3881 3891 10.1128/JB.00222-09 19376880 PMC2698405

[B16] Hoffer E Haechler H Frei R Stephan R Low occurrence of *Clostridium difficile* in fecal samples of healthy calves and pigs at slaughter and in minced meat in Switzerland J Food Prot 2010 73 973 975 20501051 10.4315/0362-028x-73.5.973

[B17] Rodriguez C Taminiau B Van Broeck J Avesani V Delmée M Daube G Clostridium difficile in young farm animals and slaughter animals in Belgium J. Anaerobe 2012 18 621 625 10.1016/j.anaerobe.2012.09.008 23041559

[B18] Zidaric V Pardon B Dos Vultos T Deprez P Brouwer MS Roberts AP Henriques AO Rupnik M Different antibiotic resistance and sporulation properties within multiclonal *Clostridium difficile* PCR ribotypes 078, 126, and 033 in a single calf farm Appl Environ Microbiol 2012 78 8515 8522 10.1128/AEM.02185-12 23001653 PMC3502897

